# Supplementary material for: The causal relationship between the human gut microbiota and pyogenic arthritis: a Mendelian randomization study
Source: Front Cell Infect Microbiol. 2024 Nov 26;14:1452480. doi: 10.3389/fcimb.2024.1452480 (PMC11629706; doi:10.3389/fcimb.2024.1452480)
Supplement: Supplementary file 1 [file DataSheet1.zip › mendelian test/ebi-a-GCST90027531.csv_leave_one_out.pdf]

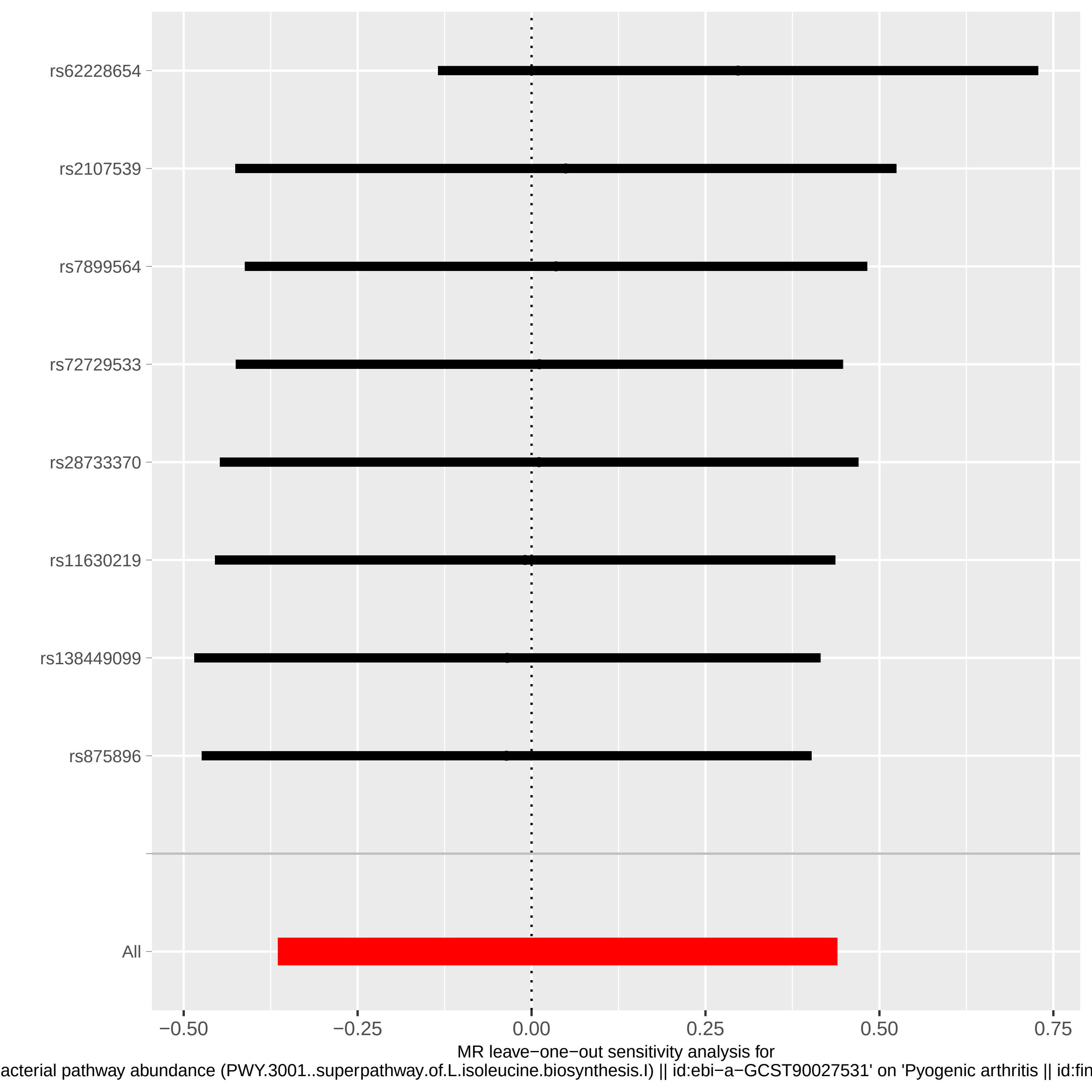
rs62228654
rs2107539
rs7899564
rs72729533
rs28733370
rs11630219
rs138449099
rs875896
All
−0.50
−0.25
0.00
0.25
0.50
0.75
MR leave−one−out sensitivity analysis for
acterial pathway abundance (PWY.3001..superpathway.of.L.isoleucine.biosynthesis.I) || id:ebi−a−GCST90027531' on 'Pyogenic arthritis || id:fin
